# Incidence of Sepsis After Transrectal Ultrasound-Guided Prostate Biopsy in Dubai Tertiary Care Hospitals: A Multicenter Cohort Study

**DOI:** 10.7759/cureus.104933

**Published:** 2026-03-09

**Authors:** Hala Nasseif, Milad Nourianpour, Nada Badawy, Mandana Gholami, Hana Mokhtar Amin, Farhad Janahi

**Affiliations:** 1 Department of General Surgery, Dubai Health, Dubai, ARE; 2 College of Medicine, Mohammed Bin Rashid University of Medicine and Health Sciences, Dubai, ARE

**Keywords:** prophylactic antibiotics, prostate biopsy, risk factors, sepsis, transrectal

## Abstract

Background: Post-biopsy sepsis is a key safety concern after transrectal ultrasound-guided prostate biopsy (TRUS-Bx), yet data from the Middle East, where antimicrobial resistance patterns and prophylaxis practices may differ, remain limited. We assessed the incidence, timing, and risk factors for post-biopsy sepsis across three tertiary care hospitals in Dubai, United Arab Emirates.

Methods: We conducted a retrospective cohort study across three tertiary care hospitals within a single private network (2010-2021; n = 139). The primary outcome was clinically recorded sepsis after TRUS-Bx; for sepsis cases, time to onset (days) was recorded. Prophylaxis regimens were coded and grouped as single-agent versus combination. Associations were summarized as odds ratios (ORs) with 95% confidence intervals (CIs) and tested using Fisher's exact tests. Continuous variables were presented as mean ± standard deviation (SD) and median (interquartile range (IQR)). Age was abstracted in categories; when a mean age was reported, category midpoints were used.

Results: The cohort was multinational (39 nationalities), with the largest groups being British (28.8%), American (8.6%), and Emirati (6.5%). Sepsis occurred in 21/138 (15.2%), averaging 1.75 cases/year. Among sepsis cases, the median time to onset was two days (IQR: 1-4; mean: 3.9; range: 1-22). Prophylaxis was documented in 133/139 patients: single-agent (58, 43.6%) versus combination (75, 56.4%). Sepsis risk did not differ by regimen group (p = 0.320) or across individual regimens (p = 0.81). Current smoking was associated with higher odds of sepsis (OR: 4.03; 95% CI: 1.31-12.39; p = 0.023), while hypertension was associated with lower odds (OR: 0.19; 95% CI: 0.053-0.69; p = 0.007). Diabetes showed no association (OR: 1.06; p = 1.00), and hyperlipidemia showed a non-significant trend (OR: 0.23; p = 0.061). Prostate-specific antigen (PSA) (n = 133) had a median of 7.74 (IQR: 5.74-15.0) and a mean of 15.70 ± 22.02. Prostate volume (n = 122) had a median of 39 mL (IQR: 30-54.8) and a mean of 45.0 ± 20.5.

Conclusion: In this multinational cohort from Dubai's private sector, post-TRUS-Bx sepsis occurred in 15.2% of cases and typically presented within the first few days. Combination prophylaxis did not reduce risk compared with single-agent regimens. Current smoking was associated with increased odds of sepsis, whereas hypertension was associated with lower odds.

## Introduction

Transrectal ultrasound-guided prostate biopsy (TRUS-Bx) remains a standard diagnostic procedure for suspected prostate cancer. Infectious complications, including sepsis, are a major concern following TRUS-Bx [1,2]. Reported sepsis incidence varies across settings and over time, influenced by local prophylaxis practices and antimicrobial resistance patterns [3-5]. Prostate cancer is a significant health concern worldwide and in the Middle East. According to the World Health Organization, prostate cancer is the second most frequent cancer among men in the United Arab Emirates (UAE) (after colorectal cancer) [6]. More than two million prostate biopsies are performed annually in the United States and Europe for cancer detection [7-9]. TRUS-Bx is generally well tolerated; common non-infectious complications include hematuria, hematospermia, and rectal bleeding [10]. However, infectious complications ranging from urinary tract infection and prostatitis to severe sepsis remain a key safety issue [11].

Empiric antimicrobial prophylaxis is standard practice to reduce post-biopsy infections [12,13]. Fluoroquinolones (typically ciprofloxacin) were long considered the mainstay because of broad-spectrum activity and prostatic penetration [14]. In recent years, the effectiveness of fluoroquinolone monotherapy has waned as fluoroquinolone-resistant *Escherichia coli *(*E. coli*) has become more prevalent [15,16]. Contemporary series report sepsis rates after TRUS-Bx of approximately 0.6-2.9% (averaging ~1.5%) despite prophylaxis [3], higher than earlier rates of <1%. A recent North American cohort reported a 2.2% infection rate despite prophylaxis [2], and some cohorts have reported rates approaching 10% [5]. Travel to regions with high antimicrobial resistance and recent antibiotic exposure are recognized risk factors for harboring resistant organisms and subsequent post-biopsy infection [17]. In parts of Europe, guidance has shifted away from routine fluoroquinolone prophylaxis toward non-fluoroquinolone or targeted alternatives and, increasingly, use of the transperineal (TP) approach to minimize infection risk.

Patient factors have also been examined. Prior studies have identified diabetes mellitus and a history of urinary tract infection or urethral catheterization as potential risk factors for post-TRUS-Bx infections [18,19], although several analyses suggest that no single patient factor conclusively predicts sepsis, with procedural and microbiologic determinants (e.g., resistant rectal flora) playing a prominent role [3]. Nonetheless, patients with significant comorbidities (e.g., diabetes or immunosuppression) are generally considered higher risk and warrant close monitoring [20].

Despite growing international attention to TRUS-Bx infectious complications, there is a paucity of data from the region, where resistance epidemiology and prophylaxis practices may differ. To our knowledge, this is the first study in Dubai to quantify the incidence of post-biopsy sepsis. Comparable regional data include a Lebanese analysis reporting urosepsis at 9.4% (range across other studies: 0.2-3.1%) [21]. In this context, we aimed (i) to estimate the incidence of sepsis after TRUS-Bx in a multinational cohort from three tertiary care hospitals within a private network in Dubai and (ii) to evaluate associations between patient-related factors (age, smoking, body mass index (BMI), and common comorbidities) and procedural factors (antibiotic prophylaxis regimens) with post-biopsy sepsis.

## Materials and methods

Design and setting

We conducted a retrospective cohort study of consecutive patients undergoing TRUS-Bx across three tertiary care hospitals, namely, Mediclinic City Hospital, Mediclinic Welcare Hospital, and Mediclinic Parkview Hospital, within a single private network in Dubai, UAE, between January 1, 2010, and December 31, 2021. Institutional ethical approval was obtained from the Dubai Scientific Research Ethics Committee of the Dubai Health Authority (approval number: DSREC-01/2023_06) prior to data collection. Patients were identified through the shared electronic medical record (EMR).

Eligibility

We included all men aged ≥18 years who underwent TRUS-Bx within our hospital network between January 1, 2010, and December 31, 2021. Procedures performed outside the network were not eligible. We excluded biopsies with clinical evidence of active urinary tract infection or prostatitis at the time of biopsy, as active infection would confound prophylaxis effectiveness. Sepsis outcome (yes/no) was available for all included patients with the exception of one patient whose data were not documented; this patient was excluded from outcome-based analyses only and retained in unrelated descriptive summaries. Analyses were based on variables contained in the study spreadsheet, and no site-level or microbiology/resistance fields were captured. For covariates with missing values, we used complete-case analysis for the relevant comparisons.

Data collection

Data were abstracted from the EMR into a predefined template by three authors. To improve data accuracy, extracted values were cross-checked against the source EMR by another author for internal consistency, and any discrepancies identified were resolved by consensus; no formal re-abstraction audit was undertaken. Variables included the following: age, abstracted from a continuous EMR field into seven predefined categories for pragmatic analysis (the 28-37-year and 88-97-year categories contained no patients and are therefore not shown in Table 1); nationality (summarized descriptively only and not included in inferential analyses); smoking (current vs no); BMI category; comorbidities (hypertension, diabetes, hyperlipidemia); serum prostate-specific antigen (PSA, ng/mL); prostate volume (mL); year of procedure; and prophylactic antibiotic regimen. Prophylaxis was routinely administered; timing and dosing were at clinician discretion at all sites, and no network-wide standardized protocol was in place during the study period. Regimen documentation was available for 133/139 patients. For antibiotic analyses, regimens were coded per a predefined key and grouped as single-agent versus combination. Additional comorbidities recorded in the EMR (e.g., gout, ischemic heart disease, asthma, renal transplant, and others) were captured and were summarized descriptively; inferential analyses were limited a priori to hypertension, diabetes, and hyperlipidemia due to event counts.

Outcome

The primary outcome was clinician-diagnosed post-biopsy sepsis (yes/no), defined as a documented infectious episode after TRUS-Bx with systemic inflammatory features (fever, chills/rigors, or hemodynamic instability) prompting emergency evaluation and/or hospital admission. Because sepsis was clinician-diagnosed from routine documentation, some inter-clinician subjectivity in ascertainment is inherent. Positive urine or blood cultures, when available in the EMR narrative, were considered supportive but were not required for case ascertainment because structured microbiology/resistance fields were not available in the dataset. For patients with sepsis, we recorded the number of days from biopsy to first sepsis presentation.

Statistical analysis

Continuous variables were summarized as mean ± standard deviation (SD) and median (interquartile range (IQR)) and categorical variables as n (%). Associations with sepsis were expressed as odds ratios (ORs) with 95% confidence intervals (CIs) from 2 × 2 tables and were tested with two-sided Fisher's exact tests. Across individual prophylaxis regimens (R × 2), heterogeneity was assessed using the Fisher-Freeman-Halton exact test due to sparse cells, and results were reported as exact two-sided p-values. Analyses used complete-case denominators, which vary by variable. Statistical significance was defined as p ≤ 0.05 (two-sided). No multivariable modeling was undertaken given 21 sepsis events relative to candidate predictors. Reporting followed the Strengthening the Reporting of Observational Studies in Epidemiology (STROBE). All analyses were performed using IBM SPSS Statistics for Windows, V. 24.0 (IBM Corp., Armonk, NY, USA). 

Study size and bias

This was a consecutive series over the study period (no a priori sample size calculation). Potential selection bias was minimized by including all eligible procedures captured in the unified EMR. We sought to reduce information bias by using predefined variable codes, a single structured spreadsheet, and author cross-checking against the source EMR; however, misclassification remains possible given reliance on routine clinical documentation. Missing data were handled by complete-case analysis; no imputation was performed.

## Results

A total of 139 patients met the inclusion criteria and underwent TRUS-Bx between 2010 and 2021. The cohort was multinational, comprising patients from 39 nationalities; the largest groups were British 40/139 (28.8%), American 12/139 (8.6%), Emirati 9/139 (6.5%), Australian 7/139 (5%), and Nigerian 7/139 (5%). Ages ranged from 38 to 87 years. The largest age bracket was 48-57 years (61/139, 43.9%), followed by 58-67 years (50/139, 36%); only 1/139 (0.7%) was aged 78-87 years. The mean prostate volume was 45.0 mL (SD: 20.5). The mean pre-biopsy PSA was 15.7 ng/mL (SD: 22.0); three entries exceeded 100 ng/mL. The median PSA was 7.74 ng/mL (IQR: 5.74-15.0). The baseline characteristics of the patients are summarized in Table 1.

**Table 1:** Demographics and baseline characteristics (n = 139) Values are presented as n/N (%) unless noted. Continuous variables are shown as mean ± SD and/or median (IQR), as available. BMI: body mass index; PSA: prostate-specific antigen; SD: standard deviation; IQR: interquartile range

Variable	Total
Age group, years	
38-47	9/139 (6.5%)
48-57	61/139 (43.9%)
58-67	50/139 (36%)
68-77	18/139 (12.9%)
78-87	1/139 (0.7%)
Smoking (n = 111)	
Current smoker	29/111 (26.1%)
Comorbidities	
Hypertension (n = 138)	57/138 (41.3%)
Hyperlipidemia/dyslipidemia (n = 138)	38/138 (27.5%)
Diabetes mellitus (n = 138)	25/138 (18.1%)
BMI (n = 118)	
18.5-24.9 (normal)	28/118 (23.7%)
25-29.9 (overweight)	52/118 (44.1%)
30-34.9 (obese)	31/118 (26.3%)
≥35 (severe obesity)	7/118 (5.9%)
Prostate volume, mL (n = 122)	45.0 ± 20.5
PSA before biopsy, ng/mL (n = 133)	15.7 ± 22.0; median 7.74 (IQR: 5.74-15.0)

Overall, 21/138 (15.2%) developed sepsis. Among sepsis cases, the median time to symptom onset was two days (IQR: 1-4; mean: 3.9, SD: 5.1; range: 1-22), and 19/21 (90.5%) occurred within the first week post-biopsy (Table 2).

**Table 2:** Sepsis outcome and timing after transrectal ultrasound-guided prostate biopsy Values are presented as n/N (%). Time to sepsis was calculated only among sepsis cases. IQR: interquartile range; SD: standard deviation

Outcome	Value
Sepsis, n/N (%)	21/138 (15.2%)
Days to sepsis (n = 21)	
Median (IQR)	2 (1-4) days
Mean (SD)	3.9 (5.1) days
Range	1-22 days

Regimen documentation was available for 133/139 (95.7%) patients (Table 3). The most common regimens were gentamicin (32/133, 24.1%), ciprofloxacin + metronidazole (28/133, 21.1%), and gentamicin + ceftriaxone (22/133, 16.5%). Single-agent prophylaxis was used in 58/133 (43.6%), and combination regimens were used in 75/133 (56.4%). Sepsis risk did not differ between combination and single-agent prophylaxis (Fisher's exact p = 0.320). Across individual regimens, sepsis rates did not differ (Fisher-Freeman-Halton exact p = 0.81).

**Table 3:** Prophylactic antibiotic regimens and associated sepsis rates (n = 133) Group comparison is single-agent vs combination (two-sided Fisher's exact test). Differences across individual regimens were assessed using the Fisher-Freeman-Halton exact test (two-sided exact p-value). One patient on gentamicin + amoxicillin/clavulanate + ciprofloxacin had missing sepsis outcome; outcome-based analyses include 74 patients with combination regimens (known outcomes) and 58 patients with single-agent regimens.

Regimen	Patients (n)	Sepsis cases (n)	Sepsis rate (%)
Combination regimens			
Ciprofloxacin + metronidazole	28	5	17.9
Gentamicin + ceftriaxone	22	3	13.6
Gentamicin + ciprofloxacin	6	2	33.3
Ceftriaxone + levofloxacin	4	1	25
Gentamicin + metronidazole + ciprofloxacin	6	1	16.7
Gentamicin + metronidazole	2	0	0
Cefazolin + gentamicin	2	0	0
Ceftriaxone + gentamicin + levofloxacin	1	0	0
Gentamicin + cefuroxime	1	0	0
Cefixime + vancomycin	1	0	0
Ciprofloxacin + trimethoprim-sulfamethoxazole	1	1	100
Gentamicin + amoxicillin/clavulanate + ciprofloxacin	1	-	-
Single-agent regimens			
Gentamicin	32	4	12.5
Levofloxacin	6	1	16.7
Ciprofloxacin	4	0	0
Cefazolin	6	0	0
Ceftriaxone	4	0	0
Metronidazole	4	1	25
Amikacin	1	0	0
Fosfomycin	1	0	0

We next analyzed potential risk factors (Table 4). These unadjusted associations are exploratory. Current smoking was associated with higher odds of sepsis (8/29 (27.6%) vs 7/82 (8.5%); OR: 4.03; 95% CI: 1.31-12.39; p = 0.023). Diabetes showed no association (4/25 (16%) vs 17/112 (15.2%); OR: 1.06; 95% CI: 0.32-3.49; p = 1.00). Hypertension was less common among sepsis cases (3/57 (5.3%) vs 18/80 (22.5%); OR: 0.19; 95% CI: 0.053-0.69; p = 0.007). Hyperlipidemia showed a non-significant trend toward lower odds (2/38 (5.3%) vs 19/99 (19.2%); OR: 0.23; 95% CI: 0.052-1.06; p = 0.061). The collapsed BMI comparison was not significant (OR: 3.03; 95% CI: 0.65-14.01; p = 0.237). 

**Table 4:** Associations with sepsis OR from 2 × 2 tables with two-sided Fisher's exact tests. Complete-case denominators applied for each comparison. Statistically significant values are denoted with *. OR: odds ratio; CI: confidence interval

Risk factor	% with sepsis among factors present	OR (95% CI)	P-value
Smoking (current)	27.6% (8/29)	4.03 (1.31-12.39)	0.023*
Diabetes mellitus	16% (4/25)	1.06 (0.32-3.49)	1.00
Hypertension	5.3% (3/57)	0.19 (0.053-0.69)	0.007*
Hyperlipidemia/dyslipidemia	5.3% (2/38)	0.23 (0.052-1.06)	0.061
BMI ≥25 (overweight/obese)	18.9% (17/90)	3.03 (0.65-14.01)	0.237

## Discussion

In this retrospective study of 139 patients undergoing TRUS-Bx in Dubai, we found an overall sepsis rate of 15.2%, which is markedly higher than rates reported in most international series [22]. Earlier literature commonly cited post-TRUS-Bx sepsis incidence around 1-3% [9]. Even contemporary studies noting an increasing trend typically report 2-6% infection rates [23]. For example, a systematic review by Loeb et al. reported sepsis in approximately 1% of biopsies in the early 2000s, rising to approximately 4% in later years [9]. A Canadian single-center study in 2018-2020 reported a 2.2% infection rate despite heterogeneous prophylaxis practices [2]. Our observed rate of 15.2% is unusually high and exceeds rates reported in neighboring Middle Eastern countries. In 2011-2013, a tertiary care center in Lebanon reported a 9.4% urosepsis prevalence despite similar empiric prophylaxis protocols [21]. In Saudi Arabia, AlKhateeb et al. reported a 5% sepsis rate among 139 patients in 2016 [24]. Our findings therefore raise concern that post-biopsy sepsis incidence in the UAE may be at the upper extreme of the reported range, potentially reflecting local antimicrobial resistance patterns or empiric prophylaxis practices during the study period. Beyond antimicrobial resistance, differences in outcome definitions, documentation practices, and healthcare utilization may have contributed to the higher observed incidence in our setting.

Several factors may contribute to the high sepsis rate observed, but our dataset does not include microbiology or resistance profiles, so we cannot attribute infections to specific pathogens or resistance patterns in this cohort. Regional antimicrobial-resistance surveillance in the UAE documents substantial resistance burdens among priority uropathogens and tracks fluoroquinolone resistance in *E. coli*, underscoring the need to tailor empiric strategies to local data [25]. In parallel, multiple studies show that fluoroquinolone-resistant rectal colonization is a key predictor of post-TRUS-Bx infectious complications and that culture-guided (rectal swab-based) targeted prophylaxis reduces infections compared with empiric regimens [26]. Across published series, the prevalence of fluoroquinolone-resistant rectal flora among men undergoing TRUS-Bx has been reported to range from approximately 6% to 48% [27], which helps explain breakthrough infections when standard fluoroquinolone prophylaxis is used. Consistent with these mechanisms, our data (Table 3) did not demonstrate a statistically significant difference in sepsis across the empiric regimens used during 2010-2021, a finding that likely reflects limited power and non-random regimen selection in routine care.

Current smoking was associated with higher odds of post-TRUS-Bx sepsis. While smoking has not been commonly reported as a risk factor in prostate biopsy studies, the association is biologically plausible. Chronic smoking can impair immune function and microvascular circulation, potentially reducing the ability to counter bacterial invasion. Smoking is recognized as a risk factor for infections across multiple organ systems [28] and has been associated with poorer outcomes in sepsis; smokers with sepsis have been reported to have approximately 1.6-fold higher mortality than non-smokers [29]. In our cohort, current smokers had approximately fourfold higher odds of developing sepsis (OR: 4.03). This association should be interpreted cautiously given the potential for unmeasured confounding; however, it suggests that current smokers may warrant enhanced counseling and closer post-procedure monitoring and should be evaluated in larger studies.

Conversely, diabetes mellitus, often cited as a risk factor for post-biopsy infection, was not associated with sepsis in our analysis. Approximately 18% of patients had diabetes, and the sepsis rate among diabetics (16%) was similar to that of non-diabetics (15%). Prior studies have shown mixed findings: some larger series have identified diabetes as a risk factor for post-TRUS-Bx hospitalization or sepsis [30], whereas others have not demonstrated a significant effect [31]. Given our sample size and event count, definitive conclusions are not possible, but diabetes did not appear to confer increased risk in this cohort.

Age was not associated with sepsis in our cohort; however, only 19/139 (13.7%) patients were aged ≥68 years, limiting the power to detect an effect. Sepsis proportions increased across BMI categories descriptively; however, the collapsed comparison (BMI ≥25 vs <25) was not statistically significant (OR: 3.03; p = 0.237). These findings are directionally consistent with reports linking higher BMI to post-biopsy infection risk (e.g., Wu et al. [23]) but should be interpreted cautiously in light of limited power.

An apparent "protective" signal was observed for hypertension and hyperlipidemia (lower sepsis proportions among those patients). We do not interpret these comorbidities as reducing infection risk; this pattern likely reflects confounding (e.g., medication effects such as statins or angiotensin-converting enzyme (ACE) inhibitors, differences in care-seeking or follow-up) and sampling variation. There is evidence that statins may modulate immune responses and potentially reduce sepsis severity or mortality [32], though not necessarily infection incidence. Clinically, a history of hypertension or dyslipidemia should not be taken to imply lower infection risk after TRUS-Bx.

With respect to antibiotic prophylaxis, our study provides real-world insight into evolving practices within a private tertiary network. Over the study period, regimens included fluoroquinolone-containing combinations (e.g., ciprofloxacin + metronidazole) as well as non-fluoroquinolone strategies (e.g., gentamicin monotherapy or gentamicin + ceftriaxone). In the post-fluoroquinolone era, some studies have reported low severe infection rates with cephalosporin-based prophylaxis regimens [33]. In our data, these practice differences did not translate into clear reductions in sepsis, likely reflecting temporal changes in resistance, non-random regimen selection, and small subgroup sizes. Empiric broadening alone may be insufficient in high-resistance settings; tailored approaches appear more effective. For example, targeted prophylaxis based on pre-biopsy rectal swab cultures has been shown to reduce infectious complications compared with empiric antibiotics [34].

Another strategy to mitigate infection risk is shifting from a transrectal to a TP biopsy approach, which avoids traversing the rectal mucosa and reduces bacterial contamination. Observational data, including a large institutional cohort, reported 0% sepsis and no hospitalizations among TP biopsy patients, compared with 2.2% sepsis in transrectal cases [35]. These findings have contributed to a global trend toward TP biopsy. During our study period, TRUS-Bx was standard at our centers; a gradual move toward TP biopsies is underway.

This retrospective design and reliance on routine documentation introduce potential misclassification and under-capture (e.g., infections managed outside the network). Six of 139 patients (4.3%) lacked a documented prophylaxis regimen, and one patient's sepsis outcome was missing within the regimen subgroup, limiting inclusion in Table 3; however, the primary outcome (sepsis yes/no) was otherwise available for the remaining cohort. With only 21 sepsis events, statistical power for subgroup analyses was limited, and multivariable modeling was not undertaken. Regimen allocation was not randomized, introducing potential selection bias; a site identifier was not captured, precluding center-level comparisons. Microbiology and resistance data were not available in structured form. Unmeasured confounding is possible because prior urinary infection, catheterization, recent antibiotic exposure or travel, and rectal colonization status were not captured, and smoking intensity was unavailable. Antibiotic dose, route, timing, adherence, and rectal antisepsis practices were not recorded. Because sepsis was clinician-diagnosed from routine documentation and Sepsis-3 variables (e.g., Sequential Organ Failure Assessment/quick SOFA (SOFA/qSOFA)) and structured microbiology fields were not available, some cases may represent febrile urinary tract infection (UTI)/prostatitis episodes labeled as sepsis, which could inflate incidence estimates. Clinical practices and resistance patterns likely evolved between 2010 and 2021, introducing time-trend confounding that we could not model. Several covariates had missingness (e.g., BMI, smoking, PSA, prostate volume), so relevant comparisons used complete-case analyses; regimen comparisons relied on small subgroups and multiple unadjusted tests, warranting cautious interpretation. Finally, post-biopsy infections managed entirely outside the network may have been missed.

Despite these constraints, this study spans 12 years across three tertiary care hospitals within a single private network and a highly multinational population, providing a real-world view of TRUS-Bx infectious outcomes in the UAE context.

## Conclusions

Post-TRUS-Bx sepsis was frequent in this Dubai cohort, and most cases presented early after biopsy. We did not observe a statistically significant reduction in sepsis with combination versus single-agent prophylaxis, and current smoking was associated with higher odds of sepsis. These findings highlight the need for locally tailored infection-prevention pathways and prospective studies with standardized definitions and microbiology data.
